# Application of Ion Exchangers with the N-Methyl-D-Glucamine Groups in the V(V) Ions Adsorption Process

**DOI:** 10.3390/ma15031026

**Published:** 2022-01-28

**Authors:** Katarzyna Burdzy, Yi-Gong Chen, Gui-Yuan Lv, Su-Hong Chen, Dorota Kołodyńska

**Affiliations:** 1 Department of Inorganic Chemistry, Institute of Chemical Sciences, Faculty of Chemistry, Maria Curie-Skłodowska University, M. Curie Skłodowska Sq. 2, 20-031 Lublin, Poland; 2 Collaborative Innovation Center of Yangtze River Delta Region Green Pharmaceuticals, Zhejiang University of Technology, Chaowang Road 18, Hangzhou 310014, China; yigongchen@hotmail.com (Y.-G.C.); chensuhong@zjut.edu.cn (S.-H.C.); 3 College of Pharmaceutical Science, Zhejiang Chinese Medical University, Hangzhou 310053, China; zjtcmlgy@163.com

**Keywords:** vanadium, ion exchangers, adsorption, Amberlite IRA 743, Lewatit MK 51, Purolite S110, Purolite S108

## Abstract

The adsorption capacities of ion exchangers with N-methyl-D-glucamine (NMDG) groups (Amberlite IRA 743, Lewatit MK 51, Purolite S110 and Purolite S108) relative to V(V) ions were tested in a batch system, taking into account the influence of various parameters, such as the adsorbent mass (0.05–0.20 g), phase contact time (1–240 min), initial concentration (10–150 mg/L), and temperature (293–333 K), as well as in a column system where the variable operating parameters were initial concentration (50, 100 mg/L), bed volume (10, 100 mL) and flow rate (0.6, 6 mL/min). Pseudo-first order, pseudo-second order, intraparticle diffusion and Boyd models were used to describe the kinetic studies. The best fit was obtained for the pseudo-second order model. The Langmuir, Freundlich and Temkin adsorption models were used to describe the equilibrium data to acquire better knowledge about the adsorption mechanism. The thermodynamic parameters were also calculated, which showed that the studied processes are endothermic, spontaneous and thermodynamically favorable. The physicochemical properties of the ion exchangers were characterized by nitrogen adsorption/desorption analyses, scanning electron microscopy (SEM), Fourier transform infrared spectroscopy (FTIR) and X-ray photo electron spectroscopy (XPS). The point of zero charge (pH_PZC_) was also determined.

## 1. Introduction

Vanadium is a transition metal relatively abundant in the earth’s crust. It is estimated that its average concentration in the upper continental crust is 97 mg/kg which significantly exceeds the contents of such common metals as nickel, copper and zinc [1]. However, its deposits are not very concentrated. In nature, it is present in crude petroleum, uranium ores, phosphate deposits and magnetites [2]. Vanadium is mainly used in the metallurgical industry as well as in chemicals, glass, ceramics, textiles, photography, and dyes.

Vanadium exists in various oxidation states in the environment, e.g., V(II), V(III), V(IV) and V(V). However, in the aquatic environment in the presence of oxygen, V(V) is the most common [3]. Unfortunately, the toxicity of vanadium compounds usually increases with the increasing oxidation state, which makes the most common form of vanadium, V(V) the most toxic [4]. The release of large amounts of vanadium ions by industry into aquatic ecosystems causes serious environmental problems, inhibiting plant growth and contaminating groundwater [5]. In addition, vanadium ions entering the human body in greater amounts can cause acute and chronic poisoning, respiratory disorders, anemia, as well as liver and kidney damage [6]. Consequently, vanadium has been included in the U.S. Environmental Protection Agency’s candidate contaminant list and the proposed minimum reporting level was set at 0.2 µg/L [7].

Depending on the concentration and pH value of the solution, vanadium forms more than 11 species of oxyanion compounds in an aqueous solution [8]. In the low pH range (<3), V(V) is present in the solution mainly as a cation VO_2_^+^. With increasing pH, there appear the anionic forms: the decavanadate species V_10_O_26_(OH)_2_^4−^, V_10_O_27_(OH)^5−^, V_10_O_28_^6−^, and mono- or polyvanadate species, e.g., VO_2_(OH)_2_^−^, VO_3_(OH)^2−^, VO_4_^3−^, V_2_O_6_(OH)^3−^, V_2_O_7_^4−^, V_3_O_9_^3−^ and V_4_O_12_^4−^ [9,10]. The presence of anionic species in such a large pH range makes the anion exchangers a potentially good method for vanadium removal from wastewaters. In addition, the adsorption method is largely effective, simple and economical, which makes it suitable for industrial applications. However, choosing the appropriate adsorbent is of great importance. Therefore, many studies have investigated different types in the process of V(V) ion removal. Kajjumba et al. [11] investigated the use of natural shale and coal wastes with amino and carbonyl functional groups as adsorbents in the vanadium adsorption process while Padilla-Rodríguez et al. [12] used the protonated chitosan flakes (PCF) (with functional amine groups) for this purpose. The paper by Naeem et al. [13] discusses the process of vanadium removal using the three commercially available metal oxide adsorbents (GTO (Dow)—TiO_2_ based and E-33 (Seven Trents), GFH (US Filter)—iron based), where the V(V) ions uptake occurs through the anion exchange mechanism, involving the formation of a binuclear bridged complex. Keränen et al. [14] used activated carbon (AquaSorb 2000), an anion exchanger (Amberjet™ 4200 Cl) with quaternary ammonium functional groups and a cation exchanger (Amberjet™ 1200 H) with sulfonate groups for the simultaneous removal of vanadium and nickel.

The aim of this study was to determine the efficiency of V(V) ion removal from aqueous solutions using ion exchangers with N-methyl-D-glucamine functional groups: Amberlite IRA 743, Lewatit MK 51, Purolite S110 and Purolite S108. These ion exchanger groups include the hydroxyl and tertiary amine groups. The *cis* position of the functional group referred to as *cis-diol* allows for an exact tetrahedral fit of, for example, boron compounds. Therefore, these ion exchangers seem to be ideal for studying the sorption process of V(V) ions. Such studies have not been described in the literature yet. As part of the research we determined the influence of the key parameters: the adsorbent mass, phase contact time, initial concentration of V(V) ions, temperature, addition of interfering ions (static method) as well as the initial concentration, bed volume and flow rate (dynamic method) on the adsorption efficiency. The physicochemical properties of the adsorbents were also investigated.

## 2. Materials and Methods

### 2.1. Ion Exchangers

Amberlite IRA 743 (IRA743) of Rohm and Haas Co. (at present the Dow Chemical Company, Philadelphia, USA), Lewatit MK 51 (MK51) of Lanxess (Cologne, Germany), Purolite S110 (S110) and Purolite S108 (S108) of Purolite International Co were used in the investigations. The specifications of these materials are as follows:

- Amberlite IRA 743—a polystyrene-divinylbezene macroporous matrix (ST-DVB) with N-methyl-D-glucamine (NMDG) groups, strongly basic, bead size 0.5–0.7 mm, total exchange capacity 0.7 meq/mL, water content 48%–54%, maximum temperature operation 348 K, pH range 0–14,
- Lewatit MK 51—a polystyrene-divinylbezene macroporous matrix (ST-DVB) with N-methyl-D-glucamine (NMDG) groups, strongly basic, bead size 0.3–1.2 mm, total exchange capacity 0.9 meq/mL, water content 40%–50%, maximum temperature operation 333 K, pH range 3–10,
- Purolite S 110—a polystyrene-divinylbezene macroporous matrix (ST-DVB) with N-methyl-D-glucamine (NMDG) groups, strongly basic, bead size 0.5–0.7 mm, total exchange capacity 0.8 meq/mL, water content 40%–50%, maximum temperature operation 333 K, pH range 0–14,
- Purolite S 108—a polystyrene-divinylbezene macroporous matrix (ST-DVB) with N-methyl-D-glucamine (NMDG) groups, strongly basic, bead size 0.4–0.6 mm, total exchange capacity 0.6 meq/mL, water content 48%–55%, maximum temperature operation 333 K, pH range 0–14.

**Figure 1:**
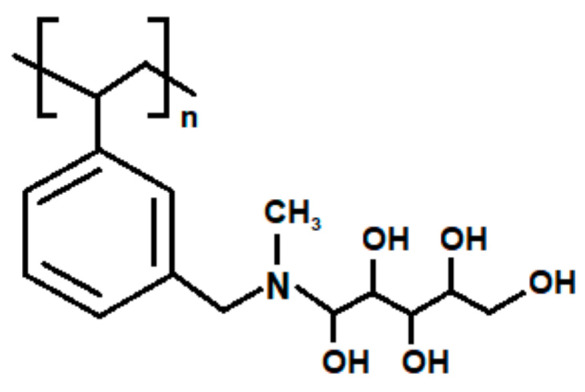
Structure of an ST-DVB ion exchanger with NMDG functional groups.

The structure of an ST-DVB ion exchanger with NMDG functional groups is presented in Figure 1:

HCl and NaOH solutions were purchased from Avantor Performance Materials, Gliwice, Poland.

In order to test the percentage of water in the tested ion exchangers IRA743, MK51, S110 and S108, the dryer-weight method was applied using the moisture analyzer MB25 (OHAUS, Parsippany, NJ, USA).

The specific surface area, the total pore and micropore volumes and the average pore diameter were calculated using the adsorption data obtained by means of the ASAP 2405 analyzer (Micromeritics Instrument Corporation, Norcross, GA, USA). Using the Brunauer–Emmett–Teller (BET) method, the specific surface area (S_BET_) was calculated. V_tot_ was then evaluated by converting the volume of nitrogen adsorbed at p/p_0_ ≈ 0.98. The micropore volume was calculated by the t-plot method.

A Quanta 3D FEG microscope with the EDS (Energy Dispersive Spectroscopy)/EBSD (Electron Backscatter Diffraction) system (FEI, Hillsboro, OR, USA) was used to find the compositional differences, topography, shape, inclination, edges, or physical differences of IRA743, MK51, S110 and S108.

Fourier transform infrared spectroscopy of IRA743, MK51, S110 and S108 was studied using the ATR-FTIR (Attenuated Total Reflectance Fourier Transform Infrared Spectroscopy) analysis instrument Cary 630 (Agilent Technologies, Santa Clara, CA, USA) the Agilent MicroLab PC software (version: B.04). The samples were placed on the diamond attachment. Each spectrum was recorded in the frequency range of 650–4000 cm^−1^ with a spectral resolution of 4 cm^−1^ and a measurement time of 30 s. The band intensities were expressed in absorbance.

907 Titrando with the dosing units (Metrohm, Herisau, Switzerland) was applied to determine the pH of the point of zero charge (pH_PZC_) by means of the pH potentiometric titration method. The instrument was set at the mode where the equilibrium pH is collected. About 100 mg of IRA743, MK51, S110 and S108 was added to the NaCl solution and placed in a container at 298 K to stay overnight for equilibrium. During the titration using NaOH or HCl, the suspension was stirred and saturated continuously with He. The experiments were carried out in the pH range of 3–10.

Thermal stability of the ion exchanger was tested by thermogravimetry using Q50 TGA (TA Instruments, New Castle, DE, USA).

The XPS measurements were made using an ultra-high vacuum multi-chamber analytical system (Prevac, Rogów, Poland). The samples were degassed at room temperature to a constant high vacuum of ~5 × 10^−8^ mbar, in the loading sluice of the UHV system. AlKα monochromatic radiation was used as a source of photoelectrons. Photoelectrons were stimulated by X-ray of a characteristic line AlKα of 1486.7 eV energy, generated by a VG Scienta SAX 100 lamp with an aluminum anode and with a VG Scienta XM 780 monochromator. The pressure in the chamber was 2 × 10^−8^ mbar during the measurements. The X-ray tube operating parameters were as follows U 12 kV, Ie 30 mA.

### 2.2. Experimental Conditions

Several conditions were studied including the effects of:

mass of ion exchanger (0.05, 0.10 and 0.20 g) for the initial concentration of V(V) 50 mg/L at pH 5.0 and contacted with 1 g/L of IRA743, MK51, S110 and S108 for 240 min at 293 K;

the phase contact time (0, 1, 5, 10, 20, 30, 60, 120 and 240 min) for the initial concentration of V(V) 10–100 mg/L at pH 5.0 and contacted with 1 g/L of IRA743, MK51, S110 and S108 at 293 K;

initial concentration (10, 25, 50, 75, 100 and 150 mg/L) at pH 5.0 and contacted with 1 g/L of IRA743, MK51, S110 and S108;

temperature (293, 313 and 333 K) for the initial concentration of V(V) 10–150 mg/L at pH 5.0 and contacted with 1 g/L of IRA743, MK51, S110 and S108 for 240 min;

interfering ions at 50 mg/L for the initial concentration of V(V) 50 mg/L at pH 5.0 and contacted with 1 g/L of IRA743, MK51, S110 and S108 for 240 min at 293 K;

desorption studies, the desorbing agents (HCl, HNO_3_, H_2_SO_4_, NH_4_OH and NaOH) at a concentration of 1 M contacted with 1 g/L of IRA743, MK51, S110 and S108 for 240 min, adsorption at an initial concentration of 100 mg/L, phase contact time 240 min, pH 5.0, temperature 293 K.

### 2.3. Column Studies

**Figure 2:**
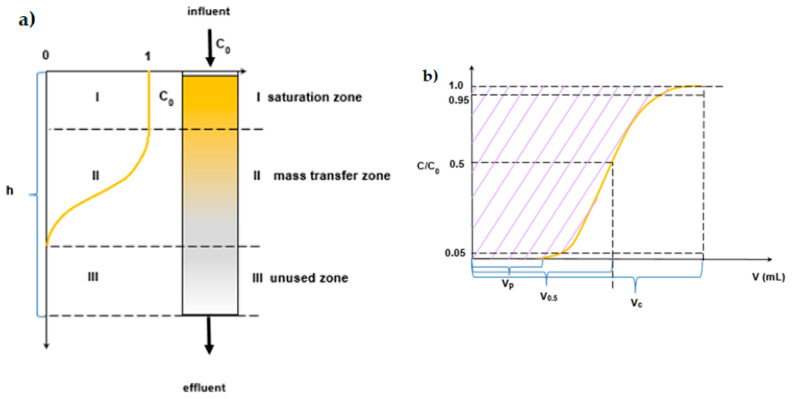

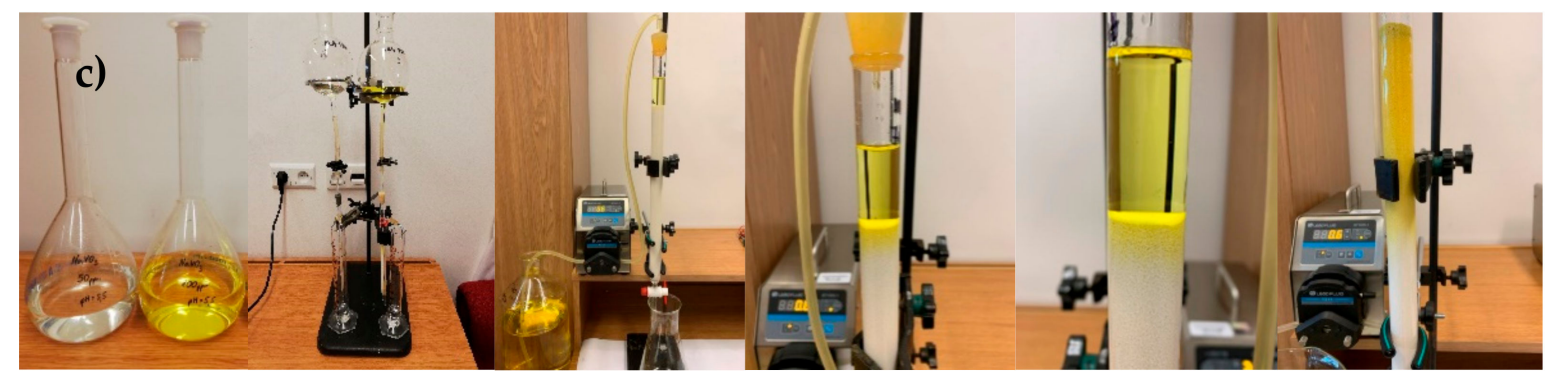
(**a**) Schematic representation of the ion exchange process in the column system, distinguishing the zones present during the process, (**b**) the breakthrough curve, (**c**) the system used in the column adsorption process.

Dynamic (column) adsorption experiments were conducted in glass columns (1.0 cm and 2.5 cm internal diameters and 20 cm and 30 cm lengths). The columns were packed with different bed volumes (10 and 100 mL) of IRA743, MK51, S110 and S108 ion exchangers between two layers of inert glass beads to prevent the floating of adsorbent particles (Figure 2).

The columns were washed with distilled water for 30 min at a constant flow rate. They were loaded with the V(V) solutions at the initial concentrations 50 and 100 mg/L. The working pH of the solution was inspected and adjusted to pH 5.5. The influent was fed by a peristaltic pump at a constant flow rate, ranging from 0.6 to 6 mL/h (Table 1). The samples were collected at regular intervals from the sampling sites.

### 2.4. Instruments

The laboratory shaker type 358A (Elpin+, Lubawa, Poland) at amplitude 7 and the stirring rate 180 rpm was used for kinetic and adsorptive studies. After the V(V) adsorption the phases were separated by means of filtration and the pH was measured using a pH meter CPI-505 with a glass-electrode (Elmetron, Zabrze, Poland). The concentration of V(V) in the solution was determined by means of the graphite furnace atomic absorption spectroscopy (GF-AAS) and the Varian AA240Z (Varian Inc., Melbourne, Australia) spectrometer with the graphite tube furnace GTA120 and the four-step time-temperature program (wavelength 318.5 nm, slit width 0.2 nm, lamp current 10 mA).

## 3. Results and Discussion

### 3.1. Ion Exchanger Characteristics

The adsorbents selected for the research are white, macroporous ion exchangers with the polystyrene matrix cross-linked with divinylbenzene with the N-methyl-D-glucamine groups. The physicochemical properties of IRA743, MK51, S110 and S108 are listed in Table 2.

**Figure 3:**
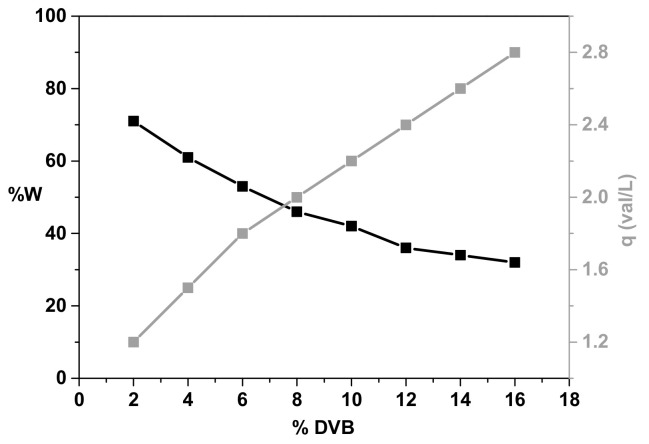
Water content depending on the degree of cross-linking and change of the capacity.

The determination of water content is important due to the possibility of an approximate assessment of the degree of cross-linking of the tested ion exchangers which is consistent with the polystyrene ion exchangers relationship presented in Figure 3 [15].

In order to test the percentage of water in the tested ion exchangers IRA743, MK51, S110 and S108, the dry weight method was used and the obtained results are summarized in Table 3.

S110 has the highest water content and MK51 the lowest. However, the obtained values are lower than those provided by the manufacturer (Table 3). Moreover, they are consistent with the preliminary estimate of the sorption capacity. In general, polystyrene ion exchangers with a higher degree of cross-linking, equivalent to the content of a cross-linking agent, e.g., divinylbenzene (% DVB), have a lower water content which indicates their better plasticity and higher capacity. Water content in the range from 24.5 to 29.7 indicates a high degree of cross-linking (about 16% DVB).

Table 4 shows the measurement results using the low-temperature N_2_ adsorption/desorption method of the tested adsorbents.

When saturated with water, the ion exchange beads have typical moisture contents from 40 to 65 wt.%. However, the more important parameter is the ion exchange capacity whose maximum value is based on the number of equivalents of mobile charge in the resin, for example 1 mol H^+^ can be equal to one equivalent (1 eq), 1 mol of Ca^2+^ is equal to two equivalents (2 eq). The dry capacity (eq/kg) is fixed but the wet capacity (eq/L) depends on the water content and the swelling degree.

The ion exchangers with high cross-linking possess the functional groups inside the microgel particles as well as on the microgel’s external surface exposed to macropores. For a higher degree of cross-linking, the number of functional groups in the micropores decreases because swelling is reduced during the functional groups’ grafting. Typically, the ion exchange materials have equilibrium exchange capacities of about 5 meq/g which depends on the amount of the functional groups. They can be grafted onto the copolymer matrix as well as on the walls of macro- or micropores. However, too many functional groups in the micropores affect the ions diffusion and the ion exchange rate slowing down. Ion diffusion in the macropores is a more complex process than in the case of gel ion exchangers.

**Figure 4:**
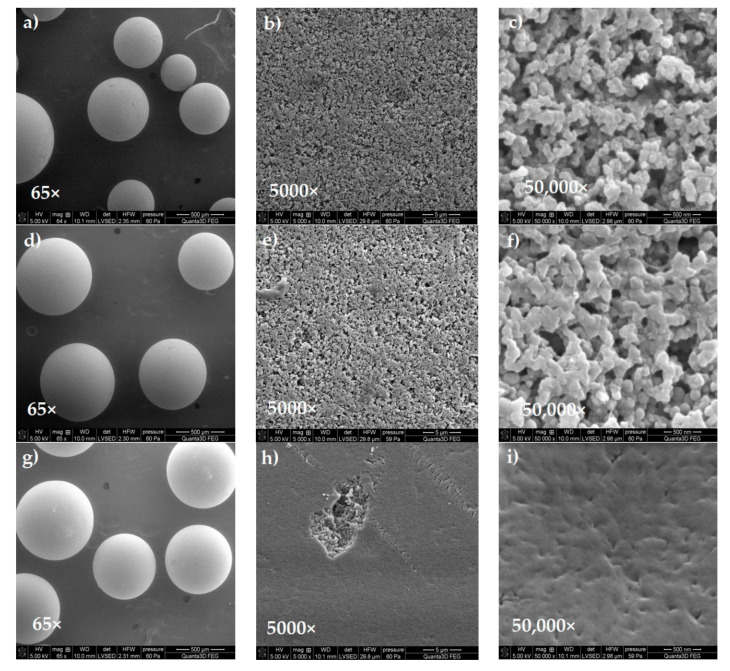

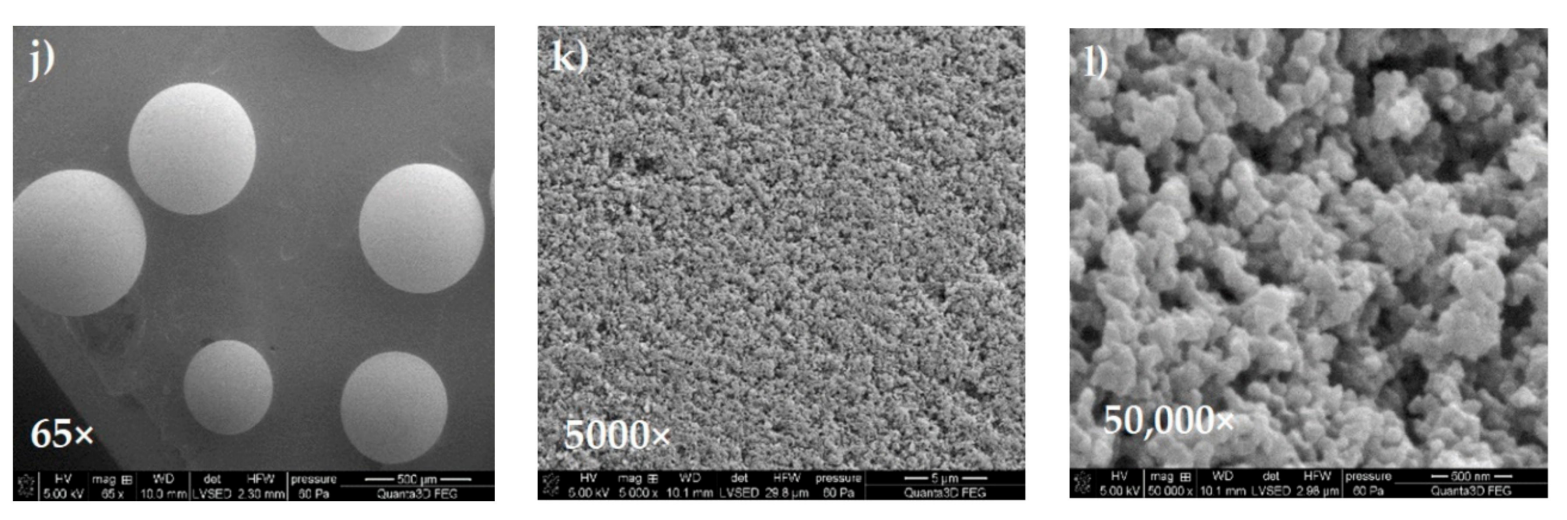
SEM images of the surface of IRA743 (**a**–**c**), MK51 (**d**–**f**), S110 (**g**–**i**) and S108 (**j**–**l**) obtained at a magnification of 65×, 5000× and 50,000×, respectively.

The Quanta 3D FEG microscope with the EDS/EBSD system (FEI) was used to find the compositional differences, topography, shape, inclination, edges, or physical differences of IRA743, MK51, S110 and S108. In order to describe the surfaces of the selected ion exchange resins, SEM images were recorded at 65×, 5000× and 50,000× magnification and they are summarized in Figure 4.

Based on the obtained SEM images, it can be concluded that the studied ion exchangers are in the form of spherical beads with a homogeneous surface. There are also visible pores in the structure of ion exchangers which are the sites for the adsorption of metal ions. Moreover, it can be seen that the S110 ion exchanger has a denser structure than the other ion exchangers.

**Figure 5:**
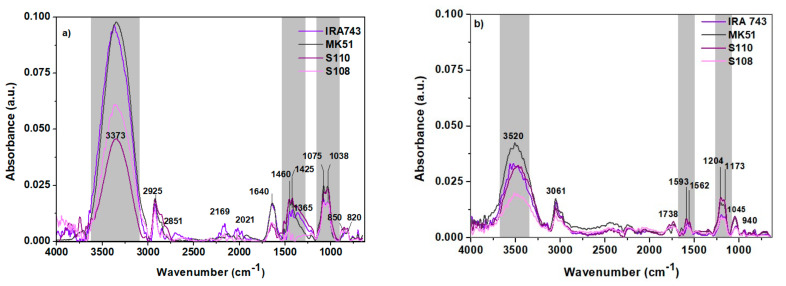
FTIR spectra of IRA743, MK51, S110, S108 before (**a**) and after (**b**) the sorption process.

The ATR-FTIR analysis was performed using the Cary 630 FTIR spectrometer by means of the attenuated total internal reflection (ATR) method. The obtained ATR-FTIR spectra are summarized in Figure 5a,b (before and after the sorption of V(V)). The assignment of the recorded vibration bands to the individual functional groups present in the molecule of the tested chemical compound with the use of vibration correlation tables made it possible to use FTIR spectroscopy to confirm the presence of the expected functional groups on the ion exchanger surface, while the analysis of the spectra after the V(V) ions sorption allowed determining the process mechanism. The position of the ATR-FTIR bands and the vibrations assigned to them from the spectra of IRA743, MK51, S110 and S108 before and after the sorption of V(V) were compared.

In the ATR-FTIR spectra of IRA743, MK51, S110 and S108 before the sorption process, broad bands of high intensity were observed at about 3373 cm^−1^. This band corresponds to the stretching vibrations of the –OH and =N–H groups. After the sorption process of V(V) ions it is reduced and shifted at the wavenumbers in the range 3480–3520 cm^−1^. A wide band in the range of 3700–3000 cm^−1^ resulting from the vibrations of the -OH groups indicates the presence of water in the ion exchanger phase. At the wavenumbers of 2925 and 2851 cm^−1^ there were observed narrow bands of high intensity attributed to the symmetric and asymmetric C–H stretching vibrations derived from the –CH_2_ group. After the sorption process, these bands were shifted without changing the intensity and are observed at the wavenumbers of 3061 and 3012 cm^−1^, respectively. They originate from the matrix of the tested ion exchangers. Bands in the wavenumber ranges 1600–1585 cm^−1^ and 1500–1400 cm^−1^ were also observed. There was also a band at a wavenumber of 1640 cm^−1^ which after the sorption process was shifted to approx. 1593 cm^−1^. These bands are characteristic of the carbon–carbon stretching skeletal vibrations inside the aromatic ring, C–H groups connected to the aromatic ring and the vibrations originating from the adsorbed water molecules. Before the sorption process the spectra, the wavenumber range 1365–1425 cm^−1^ also showed bands related to deformation vibrations in the –CH_2_ group which are characteristic of the polymer matrix of the ion exchangers. After the sorption, these bands were reduced and shifted. In turn, the bands in the range 1075–1079 cm^−1^ correspond to the stretching vibrations characteristic of the C–O groups. The bands at 850 cm^−1^ correspond to the C–C stretching vibrations in the aliphatic system and the bending vibrations of the C–H groups and at 820 cm^−1^ are attributed to strongly delocalized vibrations, including, e.g., non-planar deformation vibrations of Ar–H.

The point of zero electric charge for IRA743, MK51, S110 and S108 was determined by the potentiometric titration method. The tests were carried out for three ionic forces, in the presence of NaCl with the concentrations of 0.1, 0.01 and 0.001 M. The obtained results are summarized in Table 5.

For such molecules as V(V) which carry only negatively charged groups, the anion exchangers IRA743, MK51, S110 and S105 are the obvious choice. However, since the charge of molecules depends on pH, the choice is based on which ion exchanger (they are characterized by almost similar pH_PZC_) and pH gives the best sorption efficiency.

**Figure 6:**
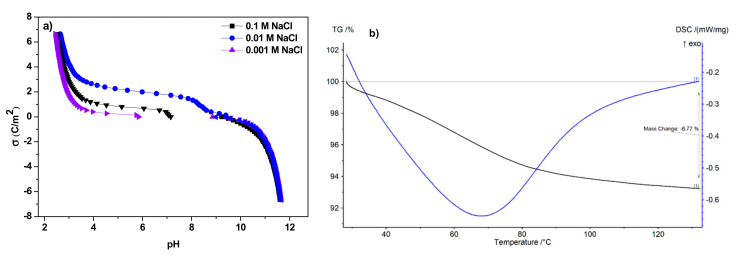
Comparison of (**a**) surface charge density of IRA743 depending on the NaCl concentration and (**b**) thermal stability of IRA743.

The surface charge densities determined as a function of pH for all ion exchangers in the NaCl solution with the concentrations of 0.1, 0.01 and 0.001 M indicate that the surface charge density decreases with the increasing solution pH. The smallest differences for the studied solutions are found for the S110 ion exchanger. Additionally, a negative surface charge occurs at pH 10.0 for the above-mentioned ion exchangers. Exemplary data are presented for the IRA743 (Figure 6a).

For most ion exchange resins, the maximum service temperatures are relatively small. They depend on the structure and ionic form of the exchanger as well as the degree of its cross-linking. Typically, for the macroporous ion exchangers, the operating temperature range is larger than for the gel ion exchangers. The thermal strength of strongly acidic cation exchangers in the hydrogen form is greater than that of medium and weakly acidic cation exchangers in the same form. In an acidic or neutral environment, the maximum allowable temperature for the use of cation exchangers should not exceed 373 K, and in an alkaline environment it should not exceed 333–353 K. Strongly basic anion exchangers in the form of OH^−^ (up to 313 K) are the least resistant to elevated temperature. On the other hand, the maximum service temperature of weakly basic anion exchangers is 333 K. Thermal stability depends also on the degree of cross-linking. However, in the case of IRA743, exemplary results are presented in Figure 6b. In the case of IRA743 above 373 K (100 °C), the TG line stabilizes, indicating that all water has been lost. The total weight loss related to the loss of water is 6%. In this case, taking into account the dynamic nature of the temperature increase, it can be expected that at the time of obtaining a stable TG curve, the total weight loss could be slightly larger.

**Figure 7:**
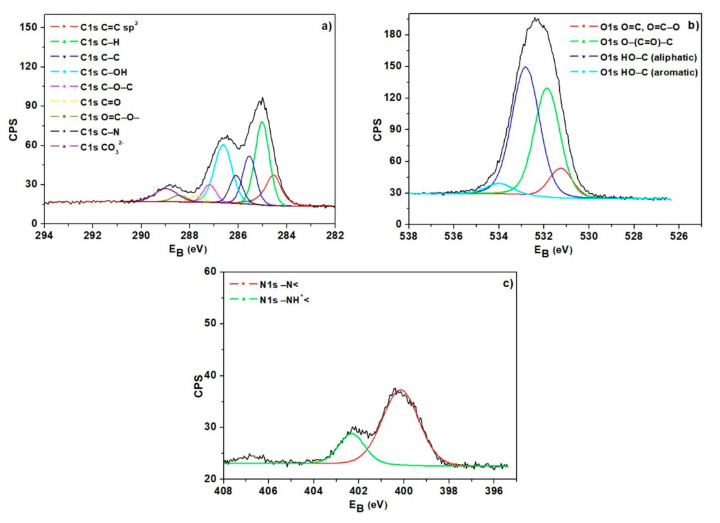
High-resolution XPS spectra of (**a**) C1s, (**b**) O1s and (**c**) N1s of IRA743.

The mechanism of V(V) Ion adsorption on the IRA743, MK51, S110 and S108 ion exchangers was determined by XPS analysis (Figure 7).

This method consists of measuring the energy distribution of electrons emitted from the sample affected by irradiation with X-rays. Due to the fact that through its surface the tested material interacts with the environment and its chemical composition determines the nature of these interactions, this method allows us to determine the type of elements included in the analyzed layer (except for hydrogen), the amounts of which are greater than 0.1% atomic fraction; the chemical state of the elements (oxidation state, form) and their mutual proportions; the chemical composition as a function of depth, and for the materials with a thickness below <100 nm, the thickness of the layer and its composition as well as whether it is homogeneous. It is known that the bond energy is influenced by the type and spatial configuration of atoms in the immediate vicinity as they affect the spatial distribution of electrons in the studied atom. The orbitals of atoms of the same element located in different chemical surroundings show slight differences in bond energy ranging from 1 up to 10 eV. In the case of XPS analysis, particular attention is paid to the spectral lines of the selected elements in order to study chemical shifts. These lines are identified by the energy of the shell from which the electrons are knocked.

Figure 7 shows the XPS spectra obtained for IRA743. The bands visible in the spectra corresponding to the levels of C1s, O1s and N1s confirm the basic elemental composition in the structure of ion exchangers. In the case of the IRA743 ion exchanger, these bands are found at the binding energies of 285.0, 531.5 and 400.0 eV for C1s, O1s and N1s, respectively. The atomic weight % was 63.4 for C, 25.9 for O, and 4.1 for N. The main forms of carbon are C=C sp^2^, C–H, C–C, C–OH and C–N, while for oxygen, the presence of the forms O=C, O=C–O, O–(C=O)–C and HO–C (aliphatic and aromatic) was proved. The high-resolution XPS spectrum of N1s indicates the presence of nitrogen mainly in the form of the amino group (–N<), as well as the protonated amino group (–NH^+^<).

### 3.2. Adsorption Studies—Static Method

#### 3.2.1. Effect of Adsorbent Mass

To determine the effect of the adsorbent mass, the adsorption process was conducted for the adsorbent mass from 0.05 to 0.20 g while the initial concentration of V(V) ions was held constant at 50 mg/L at a temperature of 293 K.

**Figure 8:**
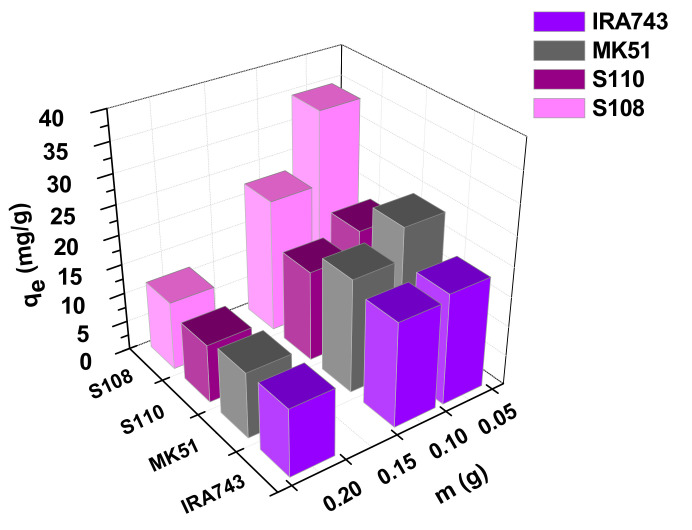
Effect of adsorbent mass on the sorption of V(V) on IRA743, MK51, S110 and S108.

Figure 8 shows the relationship between the ion exchanger mass and the obtained adsorption capacity. The sorption capacities decreased from 18.70 to 11.36 mg/g for IRA743, from 24.53 to 11.06 mg/g for MK51, from 19.23 to 9.85 mg/g for S110, and from 34.86 to 11.61 mg/g for S108 with the increase in the adsorbent mass from 0.05 to 0.20 g. This phenomenon can be explained by the large number of unsaturated adsorption sites. At the same time an increase in the percentage of V(V) ions removal is observed with the increase in the adsorbents mass (from 40.23% to 97.79% for IRA743, from 52.78% to 95.21% for MK51, from 41.37% to 84.72% for S110, and from 74.99% to 99.89% for S108) which is obviously related to the increased surface area and the active sites. Therefore, the appropriate mass of ion exchangers for further research is 0.05 g.

#### 3.2.2. Effect of Contact Time and the Kinetic Studies

**Figure 9:**
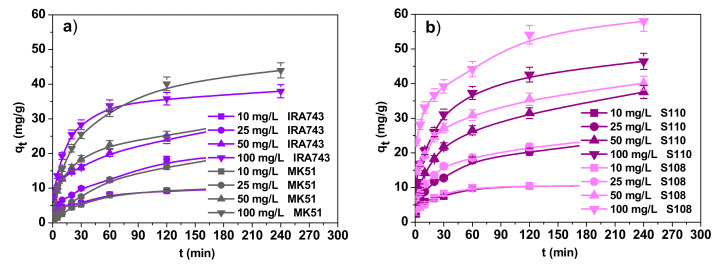
Effect of contact time and initial concentration on the removal of V(V) ions by (**a**) IRA743, MK51, (**b**) S110 and S108.

The course of the adsorption process in 240 min for different initial concentrations of V(V) ions is shown in Figure 9. It can be seen that the kinetics of V(V) ion adsorption included two stages: (1) an initial stage where adsorption is fast due to high availability of free adsorption sites and (2) a slower stage where equilibrium is achieved—the presence of adsorbed ions on the surface increases the repulsive forces, making the remaining sites more difficult to access [16]. The studies on the effect of the phase contact time proved that after about 120 min, V(V) ion adsorption equilibrium is achieved. The initial concentration affects the equilibrium establishment. The higher concentration results in a longer time period of equilibrium establishment. The obtained sorption capacities (*q_e._*_exp_) are summarized in Table 6. The *q_e._*_exp_ values correspond to the removal of V(V) ions in the amounts of 97.16%, 80.08%, 59.51% and 39.18% for IRA743; 98.86%, 83.52%, 60.40% and 45.36% for MK51; 99.91%, 95.29%, 73.50% and 47.84% for S110 and 99.81%, 97.84%, 78.49% and 59.79% for S108, for initial concentrations of 10, 25, 50 and 100 mg/L, respectively. The relationship between the initial concentration and the percentage of removed V(V) ions is clearly visible: the higher the concentration, the lower the removal percentage.

The kinetic studies and the determination of the adsorption process speed make it possible to assess the applicability of a given adsorbent in industry where fast and effective processes play a key role. For this purpose, the obtained experimental data were adjusted to the kinetic models commonly used to describe the adsorption process:

- The pseudo-first order model [17]:
  (1) $$q_{t}=q_{e}\left(1-\exp\left(-k_{1}t\right)\right)$$
- The pseudo-second order model [18]:
  (2) $$q_{t}=\frac{k_{2}q_{e}^{2}t}{1+k_{2}q_{e}t}$$
- The Weber–Morris (the intraparticle diffusion) model [19]:
  (3) $$q_{t}=k_{i}t^{1/2}+C$$
- The Boyd model [20]:
  (4) $$B_{t}=-0.4977-\ln\left(1-F\right)$$
  where: *q_e_* is the amount of V(V) ions adsorbed per amount of ion exchanger at equilibrium (mg/g), *q_t_* is the amount of V(V) adsorbed at time *t* (mg/g), *k*_1_ is the pseudo-first order rate constant (1/min), *k*_2_ is the pseudo-second order rate constant (g/mg min), *k_i_* is the intraparticle diffusion rate constant (mg/g min^1/2^), *C* is the intercept called the Weber–Morris diffusion constant, *F* is the fraction of solute adsorbed at time *t* (*F = q_t_/q_e_*).

**Figure 10:**
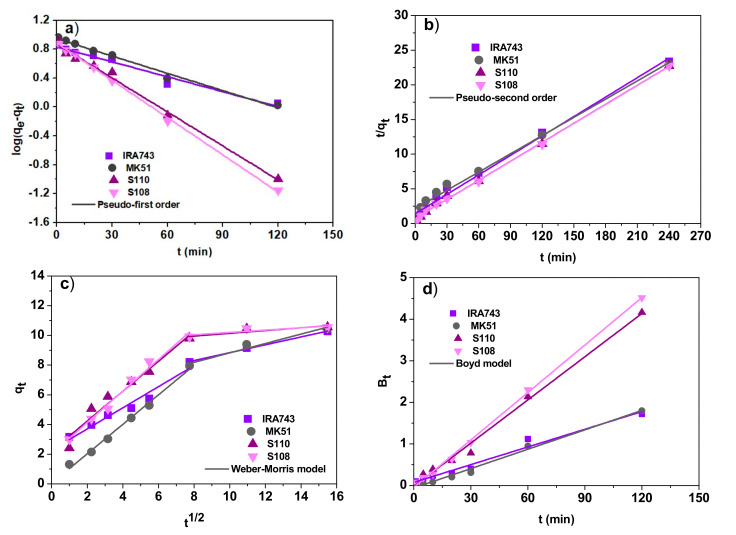
Fitting the experimental data of V(V) ions sorption on IRA743, MK51, S110 and S108 to (**a**) the pseudo-first order, (**b**) the pseudo-second order, (**c**) the Weber–Morris, and (**d**) the Boyd models (*C*_0_ = 10 mg/L).

The experimental data fitting for the V(V) ions sorption for the kinetic models at the V(V) ions concentration of 10 mg/L is shown in Figure 10. The calculated kinetic parameters for the V(V) ions concentration of 10–100 mg/L are presented in Table 6. Considering the high determination coefficients (*R^2^*), it can be concluded that the experimental adsorption kinetic data can be well described by the pseudo-second order model (*R*^2^ in the range 0.973–0.999). For the pseudo-first order model, the *R*^2^ values were slightly lower and ranged from 0.882 to 0.999. Moreover, the sorption capacities calculated on the basis of the pseudo-second order model are consistent with those obtained experimentally.

An important aspect to consider when analyzing the adsorption process is the external mass transfer. Since the pseudo-first order and pseudo-second order kinetic models cannot provide information about the rate controlling step during the V(V) ions sorption process, the Weber–Morris model was fitted to the experimental data (Figure 10c). According to this model if the plot of *q_t_* vs. *t*^1/2^ gives a straight line and passes through the origin of the coordinate system, the intraparticle diffusion is the only rate controlling step of the process, otherwise it is not the only rate limiting step due to the effect of boundary layer diffusion on adsorption. For the removal of V(V) ions on IRA743, MK51, S110 and S108, it can be seen that the obtained plots do not pass through the origin, and additionally multilinearity is revealed which indicates that apart from the intraparticle diffusion, other factors also have an influence on the adsorption rate. The confirmation of these conclusions is the application of the Boyd model where, despite the fact that the plots of *B_t_ =* −0.4977 – ln*(*1 − *(q_t_*/*q_e_))* vs. *t* (Figure 10d) have satisfactory linearity, they did not pass through the origin of the coordinate system.

#### 3.2.3. Adsorption Isotherms

Adsorption is a complex process influenced by such factors as the type and property of the adsorbent surface or the type of adsorbate [21]. Many models of adsorption isotherms are used to get to know the nature of this process. The adsorption isotherm reflects the relationship between the amount of a substance adsorbed at a constant temperature and its concentration in the equilibrium solution [22]. Many models are used to describe the adsorption process but the most common are the Langmuir, Freundlich and Temkin models. Therefore, these three models were chosen for their fitting with the experimental data obtained in the V(V) ions adsorption on IRA743, MK51, S110 and S108. For this purpose the following equations were used [23,24,25]:

- The Langmuir model [26]:
  (5) $$q_{e}=\frac{q_{0}K_{L}C_{e}}{1+K_{L}C_{e}}$$
- The Freundlich model [27]:
  (6) $$q_{e}=K_{F}C_{e}^{1/n}$$
- The Temkin model [28]:
  (7) $$q_{e}=BlnA+BlnC_{e}$$
  where *q_e_*, *C_e_* are defined as previously, *q*_0_ is the maximum amount of V(V) ions required to form a monolayer on the surface (mg/g), *K_L_* is the Langmuir constant related to the affinity for the binding sites (L/mg), *K_F_* is the Freundlich adsorption capacity (mg/g), *n* is the Freundlich constant, *A* is the Temkin constant related to the maximum binding energy (L/g), *B* is the Temkin constant related to the adsorption heat (J/mol) and it can be expressed as *B = RT/b_T_* where *R* is the gas constant (8.314 J/mol K), *T* is the absolute temperature (K) and *b_T_* is a Temkin isotherm constant.

The Langmuir isotherm model assumes that the adsorbent surface is covered with a single layer of adsorbate, and the adsorption sites are considered to be homogeneous, energetically equivalent in terms of the adsorption phenomenon, with no interactions between the adsorbed molecules [29]. The Freundlich model is used to describe the multilayer adsorption on the heterogeneous adsorbent surface while the Temkin isotherm model, unlike the two previous ones, takes into account the influence of the adsorbate–adsorbate interactions on the adsorption process and assumes that the heat of adsorption decreases linearly with the sorption coverage due to these impacts [30,31].

**Figure 11:**
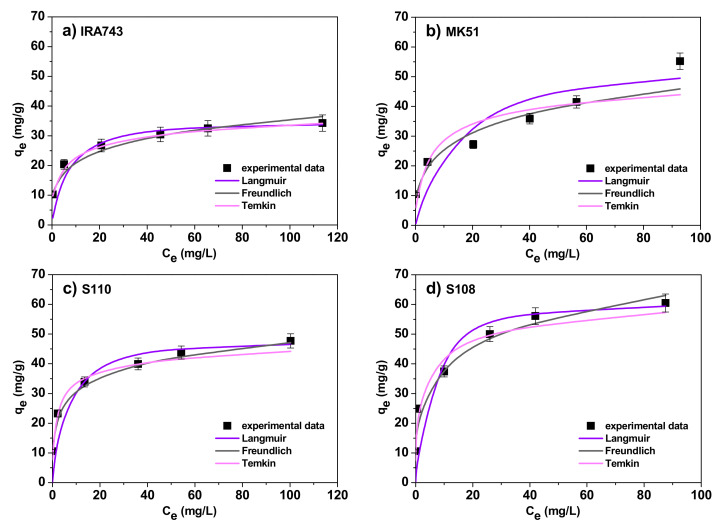
Non-linear fitting of the isotherms to the experimental data for the sorption of V(V) ions on (**a**) IRA743, (**b**) MK51, (**c**) S110 and (**d**) S108.

In order to determine the best model of the adsorption isotherm, sorption experiments of V(V) ions were conducted under the optimal conditions for initial concentrations from 10 to 150 mg/L and temperatures of 293, 313 and 333 K. Figure 11 shows the fit of non-linear forms of isotherms to the data at 293 K.

The calculated values of the adsorption isotherm parameters at various temperatures are summarized in Table 7. The comparison of the obtained values of the correlation coefficients, *R*^2^, and the chi-squared values, *χ*^2^, for the models shows that both the Langmuir and Freundlich models fit well with the experimental data (for the Langmuir model the *R^2^* values range from 0.922 to 0.997 and the *χ*^2^ values range from 0.01 to 0.70, for the Freundlich model the *R*^2^ values range from 0.958 to 0.998 and the *χ*^2^ values range from 0.01 to 1.05). A slightly worse fit was obtained for the Temkin model: the *R*^2^ values range from 0.802 to 0.996 and the *χ*^2^ values range from 0.01 to 2.89. Additionally, on the basis of the Langmuir model, the total monolayer sorption was calculated in relation to the V(V) ion sorption process carried out at different temperatures which allowed for the determination of a series of ion exchanger affinities for V(V) ions which is as follows: S108 > MK51 > S110 > IRA743. The obtained values of the *K_L_* parameter from the Langmuir model and the *K_F_* parameter from the Freundlich model confirm that the S108 ion exchanger has the highest affinity for V(V).

#### 3.2.4. Thermodynamic Study

The thermodynamic aspect of the V(V) ions adsorption on the IRA743, MK51, S110 and S108 ion exchangers was investigated to evaluate the nature of the process. For this purpose, such parameters as the change in the free energy (Δ*G°*), enthalpy (Δ*H°*) and entropy (Δ*S°*) were determined [32,33]. The change in the free energy (Δ*G*°) depends on the thermodynamic equilibrium constant *K_c_* (dimensionless) as shown by the following equation:

(8) $$∆G^{o}=-RTlnK_{c}$$

where: *R* is the universal gas constant (8.314 J/mol K), *T* is the absolute temperature (K). The equilibrium constant *K_c_* can be derived from the distribution coefficient *K_d_* (L/g) by multiplying *K_d_* by a factor of 1000.

(9) $$K_{d}=\frac{q_{e}}{C_{e}}$$

where: *q_e_* is the amount of V(V) ions adsorbed per amount of ion exchanger at equilibrium (mg/g) and *C_e_* is the V(V) ions concentration at equilibrium in solution (mg/L). The values of Δ*H°* and Δ*S°* can be estimated by the following equation:

(10) $$lnK_{c}=\frac{∆S^{o}}{R}-\frac{∆H^{o}}{RT}$$

The plot of *lnK_c_* vs. *1/T* should be a straight line. Δ*H°* and Δ*S°* values were obtained from the slope and intercept of this plot, respectively.

The calculated values of the thermodynamic parameters for the V(V) ions sorption on IRA743, MK51, S110 and S108 are presented in Table 8. The negative Δ*G°* values obtained in the study indicate that the adsorption of V(V) ions on IRA743, MK51, S110 and S108 is spontaneous and thermodynamically favorable at all tested temperatures. The Δ*G°* values were decreasing gradually with the increasing temperature, indicating that the spontaneity of the process increased with the increasing temperature. Moreover, the Δ*G°* values are in the range of −20–0 kJ/mol which may indicate a significant contribution of physical interactions in the process of V(V) ions adsorption. The additional confirmation that the interactions between the V(V) ions and the tested ion exchangers are weak are the low Δ*H°* values. The positive values of Δ*H°* indicate the endothermic nature of the process while the positive Δ*S°* values confirm the increased randomness at the solid–liquid interface during the V(V) adsorption process.

#### 3.2.5. Effect of Interfering Ions

**Figure 12:**
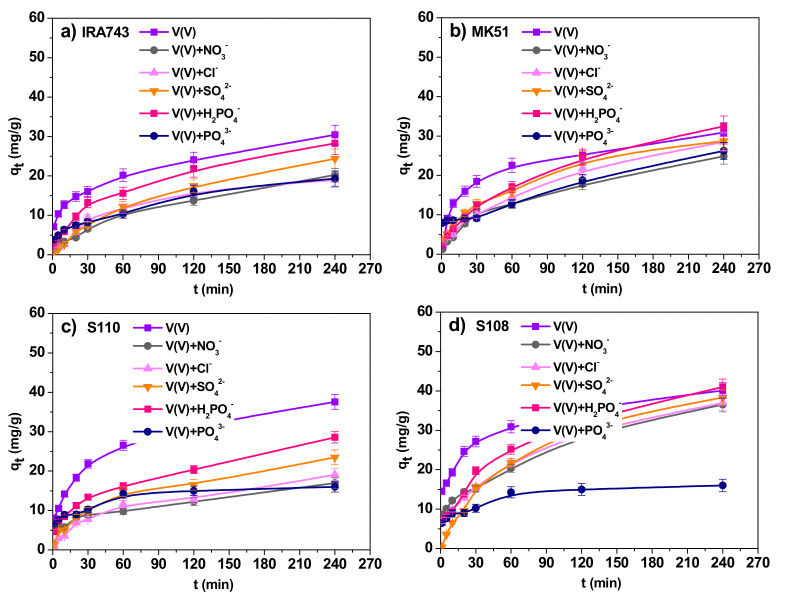
Effects of interfering ions on the V(V) ions sorption by (**a**) IRA743, (**b**) MK51, (**c**) S110 and (**d**) S108 (*C*_0_ = 50 mg/L).

The presence of additional ions in the solution can change the effectiveness of the adsorption process significantly. In order to assess the influence of interfering ions on the sorption process of V(V) ions on IRA743, MK51, S110 and S108, the adsorption studies were carried out in the presence of such ions as NO_3_^−^, Cl^−^, SO_4_^2−^, H_2_PO_4_^−^ and PO_4_^3−^. The results are shown in Figure 12.

In all tested systems, the addition of interfering ions caused changes in the process of V(V) ion adsorption. The IRA743 ion exchanger showed a decrease in the sorption capacity from 30.43 to 20.28 mg/g in the presence of NO_3_^−^ ions, to 19.00 mg/g in the presence of Cl^−^ ions, to 24.38 mg/g in the presence of SO_4_^2−^ ions, to 28.25 mg/g in the presence of H_2_PO_4_^−^ ions, and to 19.30 mg/g in the presence of PO_4_^3−^ ions. In the case of the system with the MK51 ion exchanger, smaller changes in the sorption capacities are visible: from 30.88 to 24.85 mg/g in the presence of NO_3_^−^ ions, to 28.48 mg/g in the presence of Cl^−^ ions, to 28.73 mg/g in the presence of SO_4_^2−^ ions, to 32.48 mg/g in the presence of H_2_PO_4_^−^ ions, and to 26.22 mg/g in the presence of PO_4_^3−^ ions. For the S110 ion exchanger, as for IRA743, significant decreases in the sorption capacity were observed: from 37.57 to 16.95 mg/g in the presence of NO_3_^−^ ions, to 19.08 mg/g in the presence of Cl^−^ ions, to 23.50 mg/g in the presence of SO_4_^2−^ ions, to 28.58 mg/g in the presence of H_2_PO_4_^−^ ions, and to 16.00 mg/g in the presence of PO_4_^3−^ ions. However, in the case of the S108 ion exchanger, the sorption capacity changed from 40.13 to 36.50 mg/g in the presence of NO_3_^−^ ions, to 36.98 mg/g in the presence of Cl^−^ ions, to 38.33 mg/g in the presence of SO_4_^2−^ ions, to 41.00 mg/g in the presence of H_2_PO_4_^−^ ions, and to 16.00 mg/g in the presence of PO_4_^3−^ ions. In all tested systems, the addition of PO_4_^3−^ ions had the greatest impact on the adsorption process of V(V) ions, which caused decreases in the sorption capacity of 15% in the case of MK51 up to 60% in the case of the S108. Moreover, the reduction in the speed of the process is clearly visible. The reason for such changes in the adsorption process is the fact that all tested interfering ions and V(V) are anions at the tested pH, which results in competition for active sites on the adsorbent [34].

#### 3.2.6. Desorption Studies

**Figure 13:**
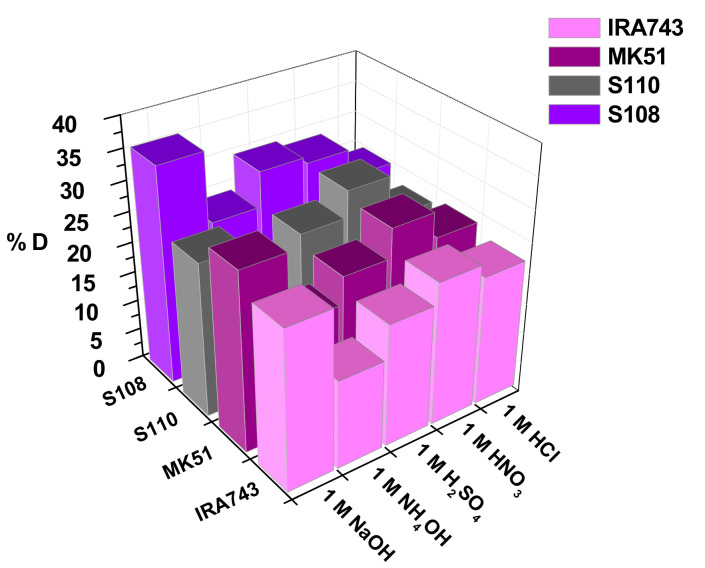
Dependence of V(V) ions desorption from IRA743, MK51, S110 and S108 using various desorption agents.

Desorption studies were carried out with the use of five desorbing agents: HCl, HNO_3_, H_2_SO_4_, NH_4_OH and NaOH at a concentration of 1 M. In the first stage, the adsorption process was carried out on the ion exchangers under the following conditions: adsorbent dose 1 g/L, initial concentration of V(V) ions 100 mg/L, phase contact time 240 min, pH 5.0, temperature 293 K. The obtained samples were dried. The desorption studies were carried out under the conditions analogous to those of the adsorption studies. The results are shown in Figure 13.

Among the tested desorbing agents, NaOH showed the best desorption ability for the IRA743, MK51 and S108 ion exchangers, while HNO_3_ was the best in the case of S110. The highest obtained desorption capacities were 25.90%, 29.15%, 28.52% and 35.62% for IRA743, MK51, S110 and S108, respectively. The obtained results of the desorption process are not fully satisfactory, which may be caused either by strong interactions between V(V) ions and ion exchangers or too little processing time.

### 3.3. Column Studies

Proper description of the ion exchange process by the dynamic method requires determination of the breakthrough curve shape and the exchange capacity for the solution under given conditions. The breakthrough curves follow the characteristic S-shaped profile produced for almost ideal adsorption systems. It is difficult to develop a model that would allow the dynamics of the ion exchange process to be accurately described as the concentration changes in the liquid and solid phases volume and time. For this reason, various mathematical models were developed to describe this process and predict the behavior of the ion exchanger during the column operation. The models used to describe the dynamics of exchange on the columns and to design and optimize this process are extremely important as they reduce the time needed to perform and repeat experiments. It is commonly known that the flow rate is one of the important parameters in the continuous treatment of wastewater effluents on an industrial scale.

In the presented studies the sorption breakthrough curves were obtained under different initial conditions such as bed volumes (10 and 100 mL), different flow rates (0.6 and 6 mL/min), initial concentration of V(V) (50 and 100 mg/L) at pH 5.0. The breakthrough curves were plotted taking into account the variation of V(V) concentration in the aqueous solution (normalized with the initial concentration of V(V)) in the function of volume effluent. The most effective system is in the case of sharp curves. The breakthrough points of the curves were determined when the V(V) concentration (*C*) reached 0.05 *C*_0_ (initial concentration). The saturation point is reached when the effluent concentration becomes equal to the influent concentration.

**Figure 14:**
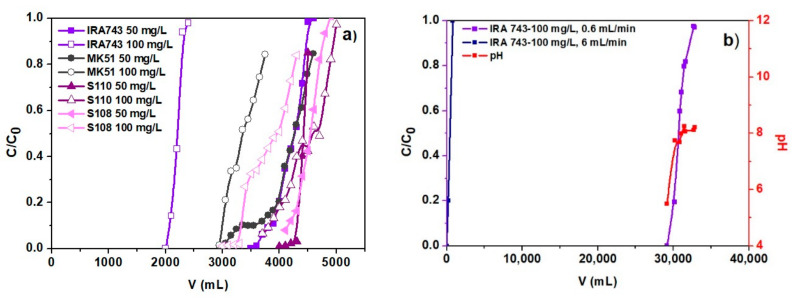
(**a**) Comparison of the breakthrough curves of V(V) ions sorption on IRA743, MK51, S110 and S108 (ID = 1 cm, *h* = 25 cm, *H* = 12–13 cm, *Q* = 0.6 mL/min, *C*_0_ = 50 and 100 mg/L), (**b**) the breakthrough curve of V(V) ions sorption and changes of solution pH during the process on IRA743 (ID = 2.5 cm, *h* = 50 cm, *H* = 25 cm, *Q* = 0.6 and 6 mL/min, *C*_0_ = 100 mg/L).

The plots of V(V) concentration in the effluent normalized to the initial concentration vs. the effluent volume are presented in Figure 14. The breakthrough curves for the initial V(V) concentrations of 50 and 100 mg/L are presented in Figure 14a for the 10 mL bed volume at a flow rate of 0.6 mL/min, and in Figure 14b for a bed volume of 100 mL. In the case of columns where the bed volume was 10 mL, the breakthrough points at which V(V) concentration reaches the value of 0.01 mg/L (corresponding to *c*/*c*_0_ 0.001) were equal to 3500 and 2000 mL for IRA743, 2950 and 2850 mL for MK51, 4000 and 3700 mL for S110 and 4100 and 3000 mL for S108, at the initial concentrations of 50 and 100 mg/L, respectively. For the system with the IRA743 ion exchanger, where the bed volume was 100 mL and the flow rate was 0.6 mL/min, the breakthrough point was 29,160 mL at the initial concentration of 100 mg/L.

The point where the V(V) concentration in the effluent reaches 0.95 of the influent is referred to as the point of exhaustion. The exhaust volumes for 10 mL bed volume were observed at 4500 and 2400 mL for IRA743, 4590 and 3750 mL for MK51, 4500 and 5000 mL for S110, 4800 and 4300 mL for S108, at initial concentrations of 50 and 100 mg/L, respectively. For IRA743 with the bed volume of 100 mL, flow rate of 0.6 mL/min and initial concentration of 100 mg/L, the exhaust volume was 32,835 mL.

The breakthrough points values increase with the increasing volume of the bed due to accessibility of more binding sites. At 10 cm bed volume, the breakthrough curves become steeper showing faster exhaustion.

The values of the important design parameters were calculated according to the equations:

- Time required for the exchange zone formation (min):
  (11) $$t_{z}=\frac{V_{E}-V_{B}}{Q}$$
- Time required for the exchange zone to become established (min):
  (12) $$t_{E}=\frac{V_{E}}{Q}$$
- Rate at which the exchange zone is moving through the bed (cm/min):
  (13) $$U_{z}=\frac{h_{z}}{t_{z}}=\frac{h}{t_{E}-t_{f}}$$
- Height of exchange zone (cm):
  (14) $$h_{z}=\frac{h\left(t_{z}\right)}{t_{E}-t_{f}}$$
- Fraction of the adsorbate present in the adsorption zone:
  (15) $$F=\frac{S_{z}}{S_{max}}=\frac{\int_{V_{B}}^{V_{E}}\left(C_{0}-C\right)dV}{C_{0}\left(V_{E}-V_{B}\right)}$$
  where: *V_E_* is the total volume to the point of exhaustion (mL), *V_B_* is the total volume to the point of breakthrough (mL), *Q* is the flow rate (mL/min), *h* is the total bed depth (cm), *t_f_* is the time required for the exchange zone to initially form *t_f_ = (1 − F)t_z_* (min), *S_z_* is the amount of solute removed by the adsorption zone from the breakthrough to exhaustion and *S_max_* is the amount of solute removed by the adsorption zone which is completely exhausted.

The capacity at the breakthrough point (*q_B_*), the capacity at the exhaustion point (*q_E_*) and the capacity from the breakthrough point to the exhaustion point (*q_EB_*) were also calculated:

- The working ion exchange capacity:
  (16) $$q_{B}=\frac{c_{0}V_{B}}{V_{j}}$$
- The capacity at the exhaustion point:
  (17) $$q_{E}=\frac{c_{0}V_{E}}{V_{j}}$$
- The capacity from the breakthrough point to the exhaustion point:
  (18) $$q_{EB}=\frac{c_{0}V_{EB}}{V_{j}}$$
  where: *V_B_* and *V_E_* are defined as previously, *V_EB_* is the volume of the eluate from the breakthrough point to the exhaustion point (mL), *c*_0_ is the initial V(V) ions concentration (mg/L) and *V_j_* is the volume of the ion exchanger in the column (mL).

The breakthrough points and the exhausted values depended on *1*/*q* (*1*/*Q*) and *H* parameters where *q* = *Q*/*S* is the superficial velocity (also known as the linear flow rate/rate of fluid flow across the unit area) (0.76 cm/min), which is the average rate of the liquid flow when the column is empty, *S* is the column section (mm^2^), *Q* is the volume flow rate (0.6 mL/min) also known as the volume velocity/fluid flow and *H* is the bed height.

The calculated values of the dynamic studies parameters for the IRA743, MK51, S110 and S108 ion exchangers under various conditions are presented in Table 9.

As follows from the research in the process of ion exchange removal of V(V) ions, the structure, chemical nature of functional groups, capacity and bead size play an important role. The ion exchangers are resins based on divinylbenzene cross-linked polystyrene. They have the same functional groups. They are macroporous. They differ in the bead size. The bead sizes are as follows: IRA743 0.5–0.7 mm, MK51 0.3–1.2 mm, S110 0.4–0.6 mm and S108 0.4–0.6 mm. Due to the different contents of functional groups, they also differ in the pH range in which they can be used. According to the manufacturer’s data, the MK51 has the smallest operating range, i.e., 3–10, and for the others it is 0–14. Analysing the breakthrough curves presented in Figure 14, it was found that all tested ion exchangers had a high affinity for V(V) ions.

The initial concentration of the solution has a significant influence on the breakthrough point. At a higher initial concentration, an early breakthrough point is observed. The total volume to the point of breakthrough increases with a decrease in the initial concentration of V(V) ions while the sorption capacity decreases. The increase in the adsorption capacity of the column with an increase in the initial concentration of V(V) ions can be explained by the fact that a high initial concentration provides a greater driving force for the mass transfer process, hence more ions can be adsorbed on the ion exchanger surface.

Comparing the results obtained for the systems with the IRA743 ion exchanger for the bed volumes of 10 and 100 mL, it can be concluded that the bed volumes affect the adsorption process. It was found that the breakthrough point increased from 2000 to 29,160 mL with increasing bed volume at a constant concentration of 100 mg/L, as expected. In addition, the working ion exchange capacity increased from 20.00 to 29.16 g/L as the bed volume increased.

## 4. Conclusions

In this study, the process of V(V) ion removal on the ion exchangers with N-methyl-D-glucamine groups (IRA743, MK51, S110 and S108) using the static method and in column systems was investigated. They are characterized by typical requirements for ion exchangers like high selectivity towards anions, high capacity to minimize the amount of needed ion exchanger, favorable kinetic and transport properties for rapid sorption, chemical and thermal stability, mechanical strength, resistance to fouling and capability of being regenerated. We investigated the influence of various process parameters: adsorbent mass, phase contact time, initial concentration of V(V) ions, temperature and the addition of interfering ions for the static method, as well as the influence of the initial concentration, bed volume and flow rate in the case of the dynamic method. It was shown that the mentioned parameters significantly affect the process of V(V) ion removal on the tested ion exchangers with the NMDG groups. Kinetic studies of V(V) ion adsorption on the IRA743, MK51, S110 and S108 ion exchangers revealed that the experimental data are well described by the pseudo-second order model. Both the Langmuir and Freundlich isotherm models fit well to the equilibrium experimental data. The obtained maximum equilibrium capacities at 293 K were 34.95, 55.79, 48.24, and 61.54 mg/g for IRA743, MK51, S110, and S108, respectively. These results indicate that the S108 ion exchanger has the greatest capability for V(V) ion removal. The analysis of the thermodynamic data indicated that the adsorption process was endothermic, spontaneous and thermodynamically favorable. The studies in the column systems showed that the adsorption capacity of V(V) ions increased with the increasing initial solution concentration and the bed volume but decreased with the increase in the flow rate.

## Figures and Tables

**Table 1:** Parameters of column studies.

Ion Exchanger	*V* (mL)	*H* (cm)	*Q* (mL/min)	*C*_0_ (mg/L)
IRA73	10 or 100	12 or 25	0.6 or 6	50 or 100
MK51	10	12	0.6 or 6	50 or 100
S110	10	13	0.6 or 6	50 or 100
S108	10	12	0.6 or 6	50 or 100

**Table 2:** Physicochemical properties of IRA743, MK51, S110 and S108.

Properties	IRA743	MK51	S110	S108
Manufacturer	Dow	Lanxess	Purolite	Purolite
Size (mm)	0.5–0.7	0.3–1.2	0.5–0.7	0.4–0.6
pH range	0–14	3–10	0–14	0–14
Max temp. (°C)	75	60	60	60
Total capacity (eq/L)	0.7	0.9	0.8	0.6
Applications	Removal of boric acid and borates from water based on the formation of a strong complex between the borate anion and the N-methylglucamine ligand	Softening, demineralization, decarbonization of industrial water and removal of nitrates from water	Chromatographic separation in pharmacology and biotechnology, catalysis (including catalytic oxygen removal)	Food industry, production of fruit juices, whey, glycerin, gelatin and sugar, evolution of boron from magnesium chloride solutions

**Table 3:** Summary of the measurement results for the percentage of water and dry mass in the ion exchangers.

Ion Exchanger	IRA743	MK51	S110	S108
%water	27.8	24.5	29.7	28.1
%dry mass	72.2	75.5	70.3	71.2

**Table 4:** Comparison of the measurement results using the low-temperature N_2_ adsorption/desorption method for IRA743, MK51, S110 and S108.

Ion Exchanger	*S_BET_* (m^2^/g)	*V_p_* (mL/g)	*V_micro_* (mL/g)	*D_p_* (nm)
IRA743	14.5	0.09	0.005	49.6
MK51	10.3	0.08	0.003	28.5
S110	12.3	0.07	0.002	27.8
S108	11.4	0.06	0.004	32.1

**Table 5:** Comparison of pH_PZC_ for IRA743, MK51, S110 and S108.

Ion Exchanger	pH_PZC_
IRA743	8.0
MK51	6.9
S110	7.8
S108	7.7

**Table 6:** Kinetic parameters for the V(V) ions sorption on IRA743, MK51, S110 and S108.

**Model**			**Pseudo-First Order**				**Pseudo-Second Order**		
**Ion Exchanger**	***C*_0_** **(mg/L)**	***q_e._*_exp_** **(mg/g)**	***q*_1.cal_** **(mg/g)**	***k*_1 _** **(1/min)**		***R*** ^**2**^	***q*_2.cal_** **(mg/g)**	***k*_2 _** **(g/mg min)**	***R*** ^**2**^
IRA743	10	10.27	6.80	0.016		0.971	10.69	0.0062	0.991
	25	20.41	17.33	0.017		0.977	22.08	0.0017	0.975
	50	30.43	20.52	0.010		0.968	31.28	0.0017	0.978
	100	38.00	22.58	0.022		0.927	39.22	0.0027	0.999
MK51	10	10.45	9.38	0.019		0.986	11.96	0.0025	0.998
	25	21.28	19.44	0.012		0.989	25.02	0.0008	0.973
	50	30.88	21.50	0.012		0.882	32.35	0.0015	0.992
	100	44.00	35.57	0.019		0.990	47.26	0.0010	0.996
S110	10	10.56	7.46	0.036		0.994	10.94	0.0113	0.998
	25	24.28	17.19	0.013		0.967	25.38	0.0021	0.987
	50	37.58	26.56	0.013		0.968	39.18	0.0014	0.988
	100	46.40	30.65	0.018		0.980	48.20	0.0016	0.996
S108	10	10.55	7.68	0.040		0.999	10.95	0.0115	0.999
	25	24.93	16.11	0.014		0.963	25.75	0.0027	0.993
	50	40.13	23.01	0.014		0.966	41.06	0.0021	0.994
	100	58.00	32.46	0.017		0.982	59.48	0.0016	0.994
		**Weber–Morris Intraparticle Diffusion**						**Boyd**	
		***k_i_*_1_** **(mg/g min^1/2^)**	***C*_1_**	***R* ^2^**	***k_i_*_2 _** **(mg/g min^1/2^)**	***C*_2_**	***R* ^2^**	***B_t_***	***R* ^2^**
IRA743	10	0.71	2.28	0.965	0.26	6.19	0.998	1.718	0.975
	25	1.32	2.29	0.995	1.00	5.60	0.873	1.764	0.947
	50	1.55	7.14	0.987	-	-	-	1.079	0.994
	100	3.81	6.26	0.968	0.54	29.73	0.995	2.351	0.951
MK51	10	0.99	0.04	0.993	0.32	5.66	0.964	1.800	0.993
	25	1.42	0.09	0.990	-	-	-	0.950	0.989
	50	2.94	1.66	0.933	1.08	13.86	0.987	1.199	0.955
	100	4.24	1.19	0.982	1.47	22.08	0.919	1.926	0.997
S110	10	1.01	2.22	0.957	0.09	9.21	0.769	4.162	0.993
	25	1.78	3.62	0.986	0.83	11.42	0.999	1.323	0.981
	50	2.87	5.04	0.989	1.42	15.67	0.999	1.326	0.994
	100	3.90	8.25	0.991	1.15	28.97	0.963	2.005	0.995
S108	10	1.06	2.01	0.986	0.08	9.42	0.751	4.518	0.998
	25	1.88	4.83	0.979	0.80	12.62	0.992	1.555	0.987
	50	2.61	11.71	0.974	1.18	22.01	0.991	1.658	0.985
	100	3.10	21.67	0.968	1.72	32.49	0.886	2.202	0.981

**Table 7:** Adsorption isotherm parameters for the V(V) ions sorption on IRA743, MK51, S110 and S108 at various temperatures.

**Model**	**-**	**Langmuir**							
**Ion Exchanger**	***T*** **(K)**	***q_e_*_.exp_** **(mg/g)**		***q*_0_** **(mg/g)**	***K_L_*** **(L/mg)**		***R*** ^**2**^		**χ^2^**
IRA743	293	34.30		34.95	0.245		0.997		0.01
	313	50.30		51.07	0.132		0.975		0.18
	333	68.50		68.27	0.122		0.940		0.70
MK51	293	55.20		55.79	0.084		0.922		0.67
	313	60.20		62.37	0.177		0.988		0.04
	333	70.00		72.51	0.150		0.975		0.15
S110	293	47.70		48.24	0.270		0.994		0.03
	313	52.30		52.90	0.320		0.995		0.02
	333	65.70		66.42	0.277		0.989		0.07
S108	293	60.50		61.54	0.317		0.993		0.02
	313	70.30		71.23	0.252		0.985		0.09
	333	76.60		77.03	0.416		0.992		0.06
**-**	**-**	**Freundlich**				**Temkin**			
**-**	**-**	***K_F_* ** **(mg/g)**	***n***	***R* ^2^**	**χ^2^**	***A*** **(L/g)**	***B*** **(J/mol)**	***R* ^2^**	**χ^2^**
IRA743	293	13.75	4.85	0.989	0.13	35.43	4.12	0.996	0.01
	313	15.52	4.03	0.997	0.08	17.32	6.03	0.936	0.67
	333	19.12	3.80	0.985	1.05	20.51	7.59	0.867	2.71
MK51	293	16.01	4.30	0.958	1.90	23.57	5.71	0.802	2.89
	313	19.06	3.84	0.996	0.01	24.14	7.24	0.924	0.41
	333	22.45	4.15	0.975	0.52	54.69	7.10	0.846	1.91
S110	293	22.00	6.05	0.996	0.01	713.62	3.95	0.935	0.28
	313	23.88	5.70	0.998	0.01	556.97	4.52	0.955	0.19
	333	26.76	4.96	0.995	0.01	273.55	5.93	0.928	0.67
S108	293	25.06	4.85	0.988	0.10	181.54	5.92	0.956	0.18
	313	26.50	4.50	0.990	0.01	127.14	6.87	0.936	0.81
	333	29.81	4.20	0.986	0.39	116.10	7.98	0.979	0.29

**Table 8:** Thermodynamic parameters for the V(V) ions sorption on IRA743, MK51, S110 and S108.

Ion Exchanger	Δ*G*° (kJ/mol)			Δ*H*° (kJ/mol)	Δ*S*° (J/mol K)
	293 K	313 K	333 K		
IRA743	−13.91	−16.25	−18.71	21.26	119.98
MK51	−15.56	−16.99	−18.82	8.27	81.15
S110	−15.02	−16.40	−18.50	10.39	86.35
S108	−15.93	−17.72	−19.32	8.94	84.96

**Table 9:** Dynamic studies parameters of V(V) ions adsorption (*Q* = 0.6 mL/min).

Ion Exchanger	IRA743			MK51		S110		S108	
*V_j_* (mL)	10		100	10					
*C*_0_ (mg/L)	50	100	100	50	100	50	100	50	100
*q_E_* (g/L)	23.0	24.0	32.8	22.9	37.5	23.0	50.0	24.5	44.0
*q_B_* (g/L)	17.5	20.0	29.2	14.3	28.5	16.5	36.0	20.0	29.0
*q_EB_* (g/L)	5.5	4.0	3.7	8.7	9.0	6.5	14.0	4.5	14.0
*D_g_*	1367.6	706.4	1018.6	1406.9	1105.7	1459.2	1449.6	1474.7	1286.6
*D_v_*	109.6	39.6	36.6	173.6	89.6	129.6	139.6	89.6	149.6
*t_f_* (min)	1805.8	627.0	5961.7	2198.2	1116.7	871.0	1920.3	1404.7	1703.3
*t_z_* (min)	1833.3	666.7	6108.3	2900.0	1500.0	2166.7	2333.3	1500.0	2333.3
*t_e_* (min)	7666.7	4000.0	54,708.3	7650.0	6250.0	7500.0	8333.3	8166.7	7166.7
*F*	0.02	0.06	0.02	0.24	0.26	0.60	0.18	0.06	0.27
*h_Z_* (cm)	4.0	2.5	3.1	6.8	3.7	4.2	4.6	2.8	5.4
*U_Z_* (cm/min)	0.0022	0.0038	0.0005	0.0023	0.0025	0.0019	0.0020	0.0019	0.0023

## Data Availability

The data presented in this study are available on request from the corresponding author.
